# Practical tips for keeping safe at work

**Published:** 2021-07-20

**Authors:** Heather Machin

**Affiliations:** 1 Project Officer: Lions Eye Tissue Donation Service, Centre for Eye Research Australia, Melbourne, Australia.

## Manual handling

Always look after your body when you are at work. This means you must be careful when you are lifting or moving an object (including a patient) and/or making repetitive movements. Here are some tips.

- Store heavier items at an appropriate height above the ground i.e. not on a high shelf or a very low shelf, which can make it unsafe for users.
- Test a load to see if it is light enough before you attempt to lift or move it.
- Always ask for help if you must move or lift an object that is heavy or difficult.
- Position yourself close to the object you want to move as this will make it easier to move.
- Wear body braces (if available), such as lifting belts.
- Do not arch your back as you move objects. Keep it straight
- Push rather than pull an item, as pushing takes less effort than pulling.
- To pick something up, bend your legs and use your stomach (core muscles) and legs to lift and push up – avoid using your back.
- Ensure you have good visibility, without adopting awkward positions, during these activities.

## Avoid repetition injury

This happens when you keep doing the same thing, in the same position, for extended periods of time; for example, people in an office sitting at a desk and typing. The key is to prevent these movements leading to strains, aches and, in some instances, severe pain. Here are some suggestions on how to prevent repetition injury:

- Take regular breaks
- Move around (stretch your limbs) between tasks – take advantage of small breaks.
- Keep repetitive motions to a minimum. Making even slight alterations to repetitive tasks can reduce the risk of injury.
- Adjust your workstation to fit the task and your individual needs (e.g., change its height)

**Figure:**
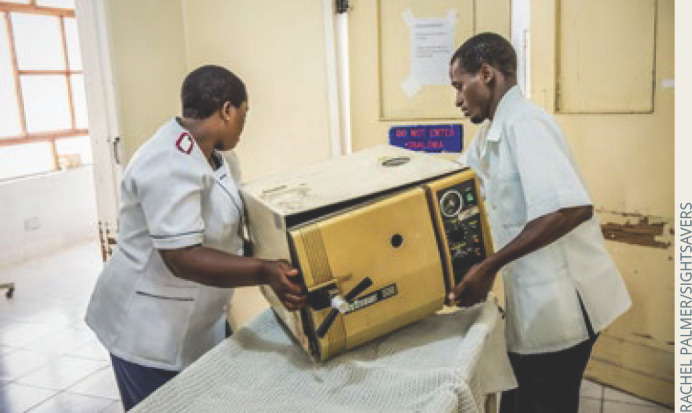
Use correct manual handling techniques to protect your back at work. **MALAWI**

## Prevent needle stick injury

Health care workers are at risk of needle stick injury and it is important to adopt safe needle-handling practices.

If you get a needle stick injury you need to immediately notify your manager and follow your hospital policy for needle-stick injuries and post-exposure prophylaxis against infection.

Here are some recommendations for prevention of needle-stick injury:

- Never re-cap a needle.
- Never take a used needle from the hand of another person. Instead, ask the person to place the sharp item into a needle container where it can be seen clearly.
- If you are the scrub nurse, never pass a needle or sharp blade to a surgeon when they are distracted, as it might harm them. Make sure you inform them that you are handing them the item so they can be alert and can safely take the item from you.
- Handle blades with a special forceps that is strong enough to grasp the blade for placement onto and off the handle’s shaft. Never use fingers.
- Only fill a sharps container to the fill line (two-thirds full).
- Never grab or stick your hand inside any bowl or container without looking first. Sharp items (i.e. suture-needles) may have been accidentally left inside.

*Adapted from Machin H. Protecting yourself at work.* Comm Eye Health Vol. 28 No. 90 2015;28-29.

